# Closed-loop model of mindfulness-based psychological regulation in competitive performance

**DOI:** 10.3389/fpsyg.2026.1794689

**Published:** 2026-04-24

**Authors:** Daliang Zhao

**Affiliations:** 1 School of Sport and Health, Guangzhou Sport University, Guangzhou, China; 2 Guangdong Province Key Laboratory of Human Sports Performance Science, Guangzhou Sport University, Guangzhou, China

**Keywords:** attentional control, Chan Buddhism, closed-loop model, mental proliferation, mindfulness-based regulation, performance

## Abstract

**Purpose:**

This study proposes a process-based, closed-loop model of mindfulness-based psychological regulation to address the lack of real-time mechanism accounts in competitive sport. The model aims to explain how athletes maintain functional operation and attentional stability under continuous performance pressure.

**Methods:**

Drawing on converging evidence from sport psychology, cognitive science, and functional principles of Chan Buddhism, the study constructs a hierarchical regulatory framework.

**Results:**

The framework establishes a hierarchical system where attentional control serves as the proximal mechanism of performance, disrupted by the core mechanism of mental proliferation. The proposed model integrates four functional dimensions (Meaning Construction, Process Orientation, Attention Anchoring, Psychological Re-centering) organized across the successive phases (initiation, propagation, execution, recovery) of the proliferation process These dimensions are dynamically coordinated through a Reference Chain (Identification-Referencing -Returning), acting as a recursive control mechanism for real-time recalibration.

**Conclusion:**

This closed-loop model shifts sport mindfulness research from outcome-based descriptions toward a process-level understanding of real-time regulation. It provides a theoretically integrative and empirically tractable foundation for enhancing performance stability in high-pressure competitive environments.

## 1 Introduction

Mindfulness-based interventions (MBIs) have gained increasing attention in sport psychology over the past two decades, with a growing body of evidence suggesting their effectiveness in enhancing both psychological wellbeing and athletic performance (Bühlmayer et al., 2017; Noetel et al., 2019; Si et al., 2024). These findings have positioned mindfulness as a promising psychological approach for athletes operating in high-pressure environments. However, despite these encouraging outcomes, the psychological mechanisms through which mindfulness facilitates performance enhancement remain insufficiently specified.

### 1.1 Limitations of existing mechanism accounts in sport mindfulness research

In the field of general psychology, several theoretical frameworks have been proposed to explain the mechanisms underlying mindfulness. For example, (Shapiro et al. 2006) proposed the Intentional Systems Model, which conceptualizes mindfulness as a process involving the interaction of three core components-intention, attention, and attitude. According to this model, the integration of these elements facilitates a process referred to as reperceiving, through which individuals alter their relationship to internal experiences. In a complementary line of work, (Vago and David 2012) developed the Self-Awareness, Self-Regulation, and Self-Transcendence framework, which explains mindfulness from a neurocognitive perspective. This framework suggests that mindfulness training promotes the development of self-awareness, self-regulation, and self-transcendence, thereby reducing cognitive biases and supporting adaptive psychological functioning. In addition, (Holas and Jankowski 2013) proposed a cognitive model emphasizing the role of mindfulness in attentional control, metacognitive awareness, and emotional processing.

These frameworks provide an important foundation for understanding the mechanisms of mindfulness and increasingly acknowledge the complexity of regulatory processes involved in mindfulness practice. However, most existing models were primarily developed within clinical psychology or general mental health contexts, where the central focus is on how mindfulness training modifies emotional responses, cognitive biases, or patterns of self-processing. In contrast, performance environments such as competitive sport involve intense situational demands in which individuals must continuously monitor attentional states, detect emerging distractions, and rapidly reorient attention toward task-relevant cues. The manner in which such real-time attentional regulation processes operate within performance contexts remains relatively under-specified in current mindfulness mechanism models. Therefore, within sport psychology, there is a need to further develop theoretical frameworks that more explicitly account for attentional regulation under performance pressure.

In the field of sport psychology, some studies have employed structural equation modeling in which variables such as rumination (Josefsson et al., 2017), self-regulation (Rogowska and Tataruch, 2024), and resilience (Qi, 2025) are frequently treated as explanatory mediators. However, these constructs primarily reflect experiential outcomes rather than functional processes directly involved in performance execution. Although, a recent meta-analysis by (Bondar et al. 2024) has demonstrated that mindfulness-based interventions are associated with reliable neural changes, however, these neural alterations were not specifically linked to performance-related cognitive and affective processes, underscoring a critical gap between mindfulness-related changes and the functional processes directly supporting performance execution.

Moreover, several mechanism-oriented frameworks in sport psychology-for example, the Mindfulness-Acceptance-Commitment (MAC) approach-suggest that mindfulness facilitates performance by promoting attentional stability, experiential acceptance, and commitment to task-relevant goals (Gardner and Moore, 2004). Empirical studies have shown that such interventions can improve athletes, emotional regulation, attentional focus, and psychological wellbeing, which in turn may contribute to more consistent performance outcomes (Noetel et al., 2019). However, despite these advances, the majority of mindfulness research in sport psychology has primarily focused on training effects and outcome variables, rather than on the real-time mechanisms through which mindfulness operates during performance. Competitive environments are inherently dynamic and unstable, often characterized by high pressure, uncertainty, and severe time constraints (Beilock and Carr, 2001; Raab, 2003; Singer, 2002). Under such conditions, psychological regulation cannot rely solely on pre-competition preparation or general improvements in emotional functioning; instead, it must operate continuously and adaptively during the performance itself (Brick et al., 2014; Gross, 2015; Oliver et al., 2020). Existing models provide limited theoretical tools for explaining how athletes regulate their mental functioning moment by moment when confronted with fluctuating internal and external demands.

### 1.2 Attentional control as a proximal mechanism of performance execution and its limitations

Within this context, attentional control has emerged as a plausible proximal mechanism linking mindfulness-related regulation to performance execution. A substantial body of sport psychology research demonstrates that the ability to allocate and sustain attention on task-relevant cues is a direct determinant of skilled performance (Wilson et al., 2009a,b). Experimental paradigms such as the Quiet Eye, attentional focus strategies, and attentional control theory consistently show that attentional stability predicts performance success, particularly under pressure (Eysenck et al., 2007; Starzak et al., 2024; Vine et al., 2013; Vine and Wilson, 2010; Wilson et al., 2009a). From a functional perspective, attentional control represents the most immediate psychological process through which internal regulation can influence action. Unlike affective or meaning-related variables such as value and acceptance-based orientations, attention directly governs information pickup, movement timing, and execution accuracy (Wulf, 2007). Therefore, identifying attentional control as a proximal mechanism provides a concrete bridge between mindfulness-related psychological processes and observable performance outcomes.

Despite their empirical strength, existing attentional control models remain insufficient for explaining psychological regulation in competitive sport. Most attentional models conceptualize attention as a relatively stable capacity or as a variable influenced by factors such as anxiety or task demands (Payne et al., 2019). Consequently, they tend to examine attention either as an outcome or as a mediator within linear causal chains (Bühlmayer et al., 2017; Josefsson et al., 2017). Such approaches offer limited insight into how attentional stability is repeatedly disrupted and restored during ongoing performance. Importantly, attentional breakdowns in competition rarely occur in isolation. They are typically embedded within broader psychological processes involving outcome concerns, self-evaluative thinking, emotional reactivity, and goal fixation (Baumeister, 1984; Hill et al., 2010; Mesagno et al., 2015). Current attentional control models provide limited theoretical structure for explaining how these processes interact over time or how athletes can disengage from disruptive mental activity and re-establish task-focused attention under pressure. As a result, attention is treated as a target of training rather than as part of a dynamic regulatory system.

### 1.3 The need for a new mechanism model

Taken together, these limitations indicate that existing mindfulness and attentional control frameworks do not fully capture the nature of psychological regulation required in competitive environments. What is needed is a mechanism model that explains not only how attention supports performance, but how athletes maintain functional psychological operation under continuous pressure. Such a model must account for the dynamic regulation of consciousness during performance, including how athletes identify emerging disruptions, recalibrate their internal orientation, and return to effective action without accumulating additional cognitive or emotional load. Developing such a model requires moving beyond technique-based explanations and toward a structurally grounded account of psychological regulation.

### 1.4 Why Chan Buddhism as a theoretical resource

Chan Buddhism differs from other Buddhist traditions primarily in its philosophy of practice rather than in specific contemplative techniques. A defining feature of Chan is its rejection of liberation as a goal to be attained through the accumulation of particular mental states or deliberate self-improvement efforts. Instead, Chan emphasizes liberation through sustained engagement with present activity without additional mental fabrication or goal fixation (Tsunoda et al., 2017). This orientation is encapsulated in the classical Chan maxim, “follow conditions to exhaust old karma (业力), and refrain from creating new afflictions (随缘消旧业, 莫再造新殃). When practice is not oriented toward attaining any particular goal, no new karmic formations are produced; as previously accumulated karmic residues are gradually exhausted, liberation naturally unfolds (Feng, 1948). From this perspective, liberation is not conceived as striving toward an ideal endpoint, but as the cessation of mental processes that perpetuate suffering while fully enacting the demands of the present situation. This practice philosophy emphasizes how consciousness relates to action under conditions of demand. The practitioner is not instructed to suppress thoughts, cultivate calmness, or generate insight as an object of pursuit; instead, regulation occurs through continuous return to the task at hand, allowing prior mental patterns to dissipate through non-reinforcement.

In competitive sport, this Chan-oriented philosophy offers a distinctive theoretical advantage. The Chan perspective directly addresses the source of disruption by targeting the mental tendency to add evaluative, anticipatory, or self-referential elaboration to ongoing action. By emphasizing full engagement with the task at hand without attachment to outcomes, Chan practice provides a conceptual framework for understanding how athletes can sustain functional awareness under pressure while minimizing the cognitive and emotional proliferation that undermines performance (Eysenck and Calvo, 1992). This orientation makes Chan Buddhism uniquely suited as a theoretical resource for modeling psychological regulation in competitive contexts. Crucially, Chan Buddhist practice does not conceptualize regulation as a one-time achievement but as an ongoing process of recognizing mental interference and returning to immediate activity. This orientation aligns closely with the demands of competitive performance, where athletes must repeatedly re-engage with task-relevant cues under pressure. By offering a process-based account of psychological functioning rather than a state-based ideal, Chan Buddhism provides a theoretically coherent foundation for developing a dynamic mechanism model tailored to competitive contexts.

It should be noted that classical Chan practices originate within a broader contemplative and religious framework aimed at spiritual realization. The present model does not attempt to reproduce this doctrinal context. Instead, it adopts a functional perspective by identifying regulatory principles embedded within these practices that may be relevant to attentional and cognitive processes during performance. In this sense, Chan concepts are used as heuristic sources for theorizing regulatory mechanisms rather than as direct psychological equivalents of contemplative techniques.

### 1.5 Purpose of the present model

Accordingly, the present study seeks to develop a novel psychological mechanism model for competitive sport by integrating principles derived from Chan Buddhist practice with contemporary sport psychology. Rather than importing religious concepts directly, the model translates core regulatory principles into a structured psychological framework designed to explain how athletes sustain effective functioning under pressure. By addressing limitations in existing mindfulness and attentional control models, this approach aims to advance a more precise and context-sensitive understanding of mindfulness-related performance enhancement in sport.

## 2 Core mechanism of the model

As one of the major traditions within Buddhist thought, Chan Buddhism differs from other schools primarily at the philosophical and practical levels. However, it shares a fundamental consensus with other Buddhist traditions regarding the three characteristics of existence—impermanence (anicca), suffering (dukkha), and not-self (anattā) (Grabovac et al., 2011). Building on this shared foundation, (Grabovac et al. 2011) translated these core Buddhist insights into contemporary psychological language and proposed a Buddhist Psychological Model to explain the mechanisms of mindfulness. Within this model, mental proliferation is identified as a central process influencing wellbeing and symptom reduction. mental proliferation is defined as a series of mental events triggered by an initial mental event or sensory impression, whereby perception becomes progressively elaborated through evaluation, interpretation, and self-referential thinking. Such proliferative processes amplify emotional reactivity and cognitive load, thereby sustaining distress and maladaptive psychological states. Conversely, reducing mental proliferation attenuates these cascades, leading to improved wellbeing and a reduction in psychological symptoms.

The model proposed in the present study likewise recognizes mental proliferation as a core psychological mechanism, but extends this framework to address the functional demands of competitive contexts. In the present model, mental proliferation refers to the rapid expansion of task-irrelevant cognitive activity during performance execution. For instance, a basketball player preparing for a decisive free throw may become preoccupied with the consequences of success or failure, such as the game's outcome or public reactions. Similarly, a tennis player might continue to mentally replay a previous unforced error during the next point instead of re-engaging with immediate tactical demands.

From a functional perspective, mental proliferation represents a central regulatory problem in competitive settings. Importantly, attention should not be conceptualized as an isolated or self-sufficient psychological process. Contemporary cognitive and sport psychology consistently emphasize that attentional allocation emerges from the dynamic interaction between top-down (goal-directed) and bottom-up (stimulus-driven) processing streams (Corbetta and Shulman, 2002; Eysenck et al., 2007; Posner and Petersen, 1989). Effective performance depends on the coordinated operation of these systems (Eysenck et al., 2007).

In competitive contexts, this coordination is highly vulnerable to disruption by mental proliferation. These proliferative processes consume limited attentional resources, bias top-down control toward maladaptive goals, and simultaneously amplify bottom-up sensitivity to threat-related or irrelevant stimuli (Eysenck et al., 2007). As a result, attention becomes fragmented and unstable, undermining its ability to remain anchored to critical performance cues. Accordingly, attentional breakdowns observed under pressure are better understood as downstream manifestations of unchecked mental proliferation, rather than as failures of attentional skill alone. This functional relationship provides the theoretical basis for positioning mental proliferation as the core regulatory mechanism that enables stable attentional control in competitive performance.

## 3 From Chan practice to functional dimensions

Chan Buddhism does not conceptualize mental training as the acquisition of specific skills, but as the transformation of how consciousness operates in the midst of action. From this perspective, mental proliferation in competition does not manifest through a single psychological channel. Instead, it unfolds through multiple functional entry points, each corresponding to a distinct mode by which consciousness becomes entangled with internal experience. Drawing on Chan practice principles, theoretical models of mindfulness training, and neuropsychological research related to mindfulness, the present model proposes four functional dimensions: Meaning Construction, Process Orientation, Attention Anchoring, and Psychological Re-centering as a minimal yet comprehensive framework capturing how proliferation is instantiated during performance execution.

### 3.1 Meaning construction: regulating value-based proliferation

In contemporary psychological models, value-based regulation has been most systematically articulated within Acceptance and Commitment Therapy (ACT), where values are conceptualized as personally endorsed principles that guide behavior across contexts (Hayes et al., 1996). Within this framework, values function to organize action and reduce experiential avoidance by providing a stable reference for behavior that is not contingent on momentary emotional or cognitive states. Importantly, values are not defined by specific outcomes, but by their role in sustaining meaningful engagement with ongoing activity (Hayes et al., 2006). This perspective highlights the regulatory function of values in shaping how individuals interpret and respond to their experiences.

Complementary insights from neuroscience suggest that value-related processing is supported by neural systems implicated in valuation and self-referential integration. For example, reflection on personally significant values has been associated with increased activity in the ventromedial prefrontal cortex, a region involved in representing subjective value and integrating self-relevant information (Falk et al., 2015). Such findings do not establish a direct neural basis for value-driven regulation in performance contexts; however, they indicate that value-based evaluative processes are grounded in identifiable neurocognitive systems that influence how information is prioritized and interpreted. In this sense, value processing may indirectly shape attentional dynamics by modulating the perceived significance of internal cognitions and external events.

Importantly, values function as stable evaluative standards, whereas meaning construction refers to the dynamic process through which such standards are applied to interpret ongoing experience. From the perspective of the present model, this modulation of perceived significance is critical in understanding the emergence of mental proliferation. In competitive environments, athletes frequently encounter performance-related events that can be rapidly imbued with evaluative meaning, such as errors, score fluctuations, or perceived expectations. When such events are interpreted in relation to personal identity, self-worth, or future prospects, they tend to trigger self-referential cognitive processing that competes for attentional resources and initiates chains of task-irrelevant thought. In this way, the amplification of meaning attribution can be understood as an upstream mechanism driving the initiation and escalation of mental proliferation. For example, when athletes associate competition outcomes with honor or their future prospects, rather than treating the competition as an activity in itself, they are more likely to experience worry. According to (Maddock et al. 2020), worry can be understood as a manifestation of mental proliferation.

A functionally convergent perspective can be found in Chan Buddhism, which emphasizes the integration of spiritual cultivation into ordinary, everyday activity rather than its separation into distinct religious or contemplative domains (Poceski, 2015). This orientation is encapsulated in the well-known Chan dictum, “chopping wood and carrying water are nothing other than the Way,” which rejects a dualistic division between action and transcendental purpose. Within this view, meaning is not externally imposed through abstract ideals or distant goals but emerges directly from wholehearted engagement with the activity at hand. From a psychological perspective, this principle can be understood as reflecting a non-attached mode of meaning construction. In a non-attached mode, meaning is grounded in engagement with the activity itself, thereby reducing the tendency for transient events to acquire disproportionate psychological significance. Functionally, it is precisely this non-attached quality of meaning construction that enables it to act as an upstream constraint on mental proliferation.

Within the present model, Meaning Construction is conceptualized as a non-attached mode of meaning construction, in which the significance of ongoing activity is anchored in the activity itself rather than in the symbolic meaning represented by its outcomes (e.g., prestige, status, or external evaluation). Compared with the conceptualization of values in ACT, non-attached meaning construction represents a further theoretical refinement at the functional level. In ACT, values are primarily understood as guiding principles that orient behavior, with their core function being to enhance behavioral consistency and reduce experiential avoidance, thereby promoting psychological flexibility.

However, the present study proposes that the critical trigger of mental proliferation lies not in the presence of values *per se*, but in the extent to which individuals attach values to specific situational events. When athletes link performance outcomes, momentary errors, or intermediate results directly to their self-worth, identity, or future prospects, the perceived significance of these events becomes amplified, thereby eliciting self-referential processing and subsequent cognitive elaboration. In contrast, non-attached meaning construction emphasizes that meaning should be grounded in value-consistent engagement with action itself, rather than derived from evaluative interpretations of specific outcomes. This shift repositions values from criteria for judging discrete performances to a stable background framework that orients action. In doing so, it constrains the emergence of meaning-driven mental proliferation at its source.

While this upstream mechanism constrains the initial amplification of significance, it does not eliminate the persistence of internally generated cognitive activity once activated, thereby necessitating additional regulatory processes at the level of ongoing cognition.

### 3.2 Process orientation: regulating outcome-oriented proliferation

In sport and performance psychology, the importance of process-oriented engagement has been articulated across multiple theoretical frameworks. Within achievement goal theory, a distinction is drawn between task-oriented and ego-oriented forms of involvement, with the former emphasizing mastery and skill execution, and the latter emphasizing normative comparison and outcome evaluation (Nicholls, 1984). Similarly, goal-setting theory differentiates between process goals and outcome goals, demonstrating that goals focused on task execution facilitate attentional stability and performance consistency, particularly under pressure (Locke and Latham, 2002). From a motivational perspective, these frameworks converge in suggesting that directing cognition toward the unfolding process of action reduces susceptibility to evaluative distraction.

A complementary line of research from cognitive and performance science provides further insight into the mechanisms underlying this distinction. Attentional Control Theory posits that anxiety-related cognition, particularly worry, consumes working memory resources and impairs goal-directed attentional control (Eysenck et al., 2007). Research on choking under pressure similarly indicates that performance breakdowns are often associated with shifts of attention toward outcome-related concerns or self-monitoring processes, which interfere with the automatic execution of well-learned skills (Beilock and Carr, 2001).

These findings point to a common functional mechanism: outcome-oriented cognition does not merely coexist with task demands, but can evolve into sustained cognitive elaboration that competes with task-relevant processing. Within the present framework, this elaborative process constitutes a central pathway through which mental proliferation unfolds during performance. Once activated, evaluative thoughts-such as concerns about results, expectations, or consequences-may trigger recursive chains of internally generated cognition, thereby increasing cognitive load and reducing the efficiency of attentional allocation.

A functionally convergent perspective can be found in Chan Buddhist teachings, which emphasize non-abiding awareness and the relinquishment of fixation on past, present, and future mental contents. The Diamond Sutra teaches that “the past mind cannot be grasped, the present mind cannot be grasped, and the future mind cannot be grasped,” articulates a mode of engagement in which cognition is not allowed to fixate on evaluative or temporally displaced content. Rather than eliminating the occurrence of thoughts, this perspective highlights the importance of preventing their continued elaboration. Although originating from a distinct philosophical context, this orientation aligns functionally with cognitive accounts of performance disruption by emphasizing the regulation of ongoing mental activity rather than its initial activation.

Within the present model, Process Orientation is defined as the capacity to sustain engagement with the unfolding process of performance while constraining the propagation of outcome-related and evaluative cognition. Importantly, this dimension does not eliminate goal representations or evaluative awareness; instead, it limits the extent to which such representations develop into sustained cognitive elaboration. In this sense, Process Orientation operates as a regulatory mechanism at the level of cognitive propagation. Functionally, this dimension occupies an intermediate position within the broader regulatory system. Whereas, Meaning Construction constrains the initial assignment of excessive significance to performance events, Process Orientation limits the continuation and amplification of cognitive activity once such significance has been activated.

### 3.3 Attention anchoring: regulating perceptual proliferation

In mindfulness-based approaches to sport performance, the regulation of present-moment attention has been identified as a core mechanism. For instance, the Mindfulness-Acceptance-Commitment (MAC) approach conceptualizes mindfulness as involving sustained, non-judgmental attention to present-moment experience, enabling athletes to remain engaged with task-relevant information during performance (Gardner and Moore, 2004). Within this framework, attentional focus is not merely a passive state, but an actively maintained orientation that allows performers to resist distraction from both internal and external sources.

Within the mindfulness literature, present-moment attention is often further differentiated into two complementary modes: focused attention and open monitoring (Lutz et al., 2015). Focused attention involves sustaining attention on a specific object or cue, whereas open monitoring involves a non-selective awareness of ongoing experience without fixation on any particular element (Lutz et al., 2008). While both modes are relevant to contemplative practice, the demands of competitive sport place particular emphasis on focused attention, as effective performance requires the selective prioritization of task-relevant information under time constraints. Empirical research in sport psychology strongly supports the importance of focused attentional engagement. Studies using the Quiet Eye paradigm demonstrate that expert performers exhibit longer and more stable fixations on task-relevant targets prior to movement execution, whereas attentional instability is associated with performance errors (Vickers, 2007). Similarly, eye-tracking research in dynamic environments indicates that successful performance depends on the efficient selection and maintenance of attention on critical perceptual cues, such as opponent movements or object trajectories (Davids et al., 2005; Williams and Davids, 1998). These findings suggest that attentional stability at the perceptual level is a key determinant of execution quality.

From a functional standpoint, such stability reflects the integrity of perception–action coupling. Effective performance depends on the continuous alignment between attentional processes and the perceptual information that directly guides motor execution (Davids et al., 2012). When attention is stably anchored to task-relevant cues, perceptual input can be efficiently translated into coordinated action. Conversely, when attention becomes fragmented or is captured by irrelevant stimuli, this coupling is disrupted, leading to delays, inaccuracies, or breakdowns in motor execution.

Within the present framework, such disruptions are conceptualized as perceptual proliferation. Unlike cognitive proliferation, which involves internally generated chains of thought, perceptual proliferation refers to the uncontrolled dispersion of attention across competing stimuli at the perceptual level. This may be driven by bottom-up factors, such as salient environmental changes, or by top-down influences, such as residual cognitive interference. In either case, the result is a breakdown in the stability of attentional allocation, undermining performance execution.

A functionally convergent perspective can be found in Chan Buddhist practice, particularly in techniques such as “kan huatou.” “Kan” means “to watch closely,” and “hua tou” refers to the point before language and thought arises. “Kan huatou” refers to focusing on the beginning of a phrase, preventing it from fully forming, which emphasize sustaining awareness at a pre-conceptual level prior to the emergence of discursive thought. Although traditionally embedded in contemplative inquiry, the functional structure of this practice highlights the stabilization of attention at an immediate experiential reference point, thereby reducing susceptibility to both internal distraction and external attentional capture. Interpreted in psychological terms, this reflects a form of attention anchoring in which awareness remains continuously coupled to a stable point of reference.

Within the present model, Attention Anchoring is defined as the capacity to maintain a stable coupling between attention and performance-relevant perceptual cues during execution. These cues may be externally oriented (e.g., opponent behavior, environmental dynamics) or internally oriented (e.g., proprioceptive feedback, movement rhythm), depending on task demands. Functionally, this dimension constrains perceptual proliferation by limiting the dispersion of attention across irrelevant stimuli and preserving the integrity of perception–action coupling.

In relation to the broader regulatory system, Attention Anchoring operates at the level of real-time perceptual regulation. Whereas, Process Orientation constrains the propagation of task-irrelevant cognitive elaboration, Attention Anchoring stabilizes attentional allocation during execution, preventing both top-down and bottom-up sources of interference from disrupting performance. In doing so, it provides a critical foundation for sustained task engagement and supports rapid recovery through Psychological Re-centering when attentional disruption occurs.

### 3.4 Psychological re-centering: recovery and restoration of task engagement

Within contemporary mindfulness research, attentional regulation is increasingly conceptualized as a dynamic process involving not only the maintenance of focus but also the detection and correction of attentional lapses. Models of focused-attention meditation describe a recurrent cycle in which attention is initially sustained on a target, followed by mind wandering, subsequent awareness of deviation, disengagement from task-irrelevant processing, and the re-establishment of attention (Lutz et al., 2008). This cyclical structure highlights that attentional stability is not defined by the absence of distraction, but by the efficiency with which deviation is detected and corrected. Empirical findings provide converging support for the functional importance of such recovery processes. (Hasenkamp et al. 2012) have demonstrated that focused-attention meditation involves a sequence of cognitive phases, including mind wandering, awareness of attentional drift, shifting of attention, and renewed sustained focus. Parallel evidence in sport and performance contexts indicates that effective performance depends on the ability to restore attentional engagement following disruption. For example, research on instructional self-talk has shown that athletes can rapidly reorient attention toward task-relevant cues after performance errors or lapses (Hatzigeorgiadis et al., 2011). These findings suggest that performance regulation is not solely dependent on sustained attention, but critically relies on the capacity to recover functional cognitive alignment under dynamic conditions.

From a functional perspective, cognitive and affective proliferation often emerges following performance-relevant perturbations, such as errors, unexpected outcomes, or physiological arousal. Once triggered, such proliferation may evolve into cascades of self-referential thought, evaluative processing, or internally generated distraction, thereby competing with task-relevant cognitive resources. The critical regulatory challenge, therefore, lies not only in preventing the emergence of such processes, but in interrupting their continuation once initiated and restoring task-directed engagement.

A functionally convergent account can be found in Chan Buddhist practice, which explicitly acknowledges that attentional deviation is inevitable and emphasizes the manner of recovery rather than the prevention of distraction. Practices such as “kan huatou” inherently incorporate both the recognition of attentional drift and the deliberate return to a point of focus. Rather than striving for uninterrupted concentration, Chan-based regulation cultivates a continuous cycle of detection and return, highlighting the importance of flexible re-adjustment in response to ongoing mental activity.

Within the present model, Psychological Re-centering is defined as the capacity to detect deviations in cognitive and affective processing, disengage from emerging mental proliferation, and rapidly restore alignment with task-relevant performance processes. This dimension does not eliminate affective responses or cognitive activation; instead, it constrains their continuation by preventing the formation of extended cognitive or behavioral consequences. In this sense, Psychological Re-centering operates as a recovery mechanism that interrupts ongoing proliferation and re-establishes functional engagement.

In relation to the broader regulatory system, Psychological Re-centering occupies a temporally distinct role. Whereas, Meaning Construction constrains the initial assignment of significance, Process Orientation limits the propagation of cognitive elaboration, and Attention Anchoring stabilizes perceptual engagement, Psychological Re-centering ensures that, when disruption inevitably occurs, the system can rapidly return to an optimal functional state. It therefore represents a critical mechanism for maintaining performance stability under fluctuating internal and external demands.

## 4 Reference chain: a Chan-based closed-loop of psychological regulation

The Buddhist Psychological Model developed by (Grabovac et al. 2011) proposes that core elements such as attention regulation, acceptance, and mental proliferation are interconnected through feedback loops, whereby changes in one component recursively influence others. These feedback loops illustrate that reductions in mental proliferation can enhance attentional clarity, which in turn improves the capacity to detect further attentional lapses, thereby reinforcing ongoing regulatory processes. This perspective provides a useful conceptual foundation for understanding psychological regulation as a dynamically self-adjusting system.

Building on this framework, the present model extends the notion of feedback loops to the domain of competitive performance by specifying how mental proliferation emerges and is regulated in real time. As outlined in Sections 3.1–3.4, mental proliferation can be conceptualized as a temporally evolving process involving (a) the assignment of subjective significance to performance events, (b) the propagation of task-irrelevant cognitive activity, (c) the destabilization of attentional allocation, and (d) the necessity for recovery following disruption. These stages are not independent; rather, they form a cascading process in which earlier phases increase the likelihood of subsequent disturbances.

Within this dynamic system, the four regulatory dimensions identified in the present model operate at different points along the proliferation cascade while also interacting recursively. Meaning Construction constrains the initial amplification of significance, thereby reducing the probability that performance events trigger downstream disruption. Process Orientation limits the continuation of internally generated cognitive elaboration, preventing the escalation of task-irrelevant processing. Attention Anchoring stabilizes the allocation of attentional resources at the perceptual–motor level, reducing susceptibility to both internally and externally driven distraction. Psychological Re-centering, in turn, enables the interruption of ongoing proliferation and the restoration of functional task engagement following disruption. Importantly, improvements in any one of these dimensions can generate downstream effects that feed back into earlier stages of the process. For example, reduced cognitive elaboration enhances attentional stability, which in turn facilitates more efficient detection of subsequent deviations, thereby strengthening the overall regulatory system.

To capture the dynamic coordination among these components, the present model introduces the concept of a reference chain as the core operational mechanism of this closed-loop system. The reference chain consists of three interrelated processes: Identification, Referencing, and Returning. Identification refers to the detection of mental proliferation or attentional deviation as it emerges during performance. Referencing involves orienting attention toward a stable regulatory anchor, which may take the form of a task-relevant perceptual cue, a process-oriented focus, or a value-consistent interpretative frame. Returning denotes the re-establishment of functional engagement with the ongoing performance task. Crucially, this sequence operates iteratively rather than linearly. Under competitive conditions characterized by continuous fluctuation, attentional deviation and cognitive interference are not exceptional events but recurrent features of performance. The effectiveness of regulation therefore depends on the efficiency with which the system cycles through identification, reorientation, and return. Each successful cycle not only restores immediate task engagement but also enhances the system's sensitivity to future deviations, thereby strengthening subsequent regulatory responses. In this sense, the reference chain constitutes a feedback-driven control mechanism that maintains performance stability through continuous monitoring and adjustment.

At the functional level, this closed-loop structure conceptualizes psychological regulation as an ongoing process in which regulatory operations and psychological states mutually influence each other over time. This formulation accounts for the inherently unstable and rapidly changing nature of competitive environments, in which effective functioning depends on the capacity for continuous recalibration rather than static control.

## 5 Hierarchical organization of the regulatory mechanism

The present model adopts a hierarchical organization to conceptualize psychological regulation in competitive contexts as a closed-loop system spanning from distal regulatory orientations to proximal performance execution. Within this framework, regulation is structured into a distal regulatory system—comprising Meaning Construction (initiation), Process Orientation (propagation), Attention Anchoring (execution), and Psychological Re-centering (recovery)—alongside core and proximal mechanisms that operate during real-time performance.

**Figure 1:**
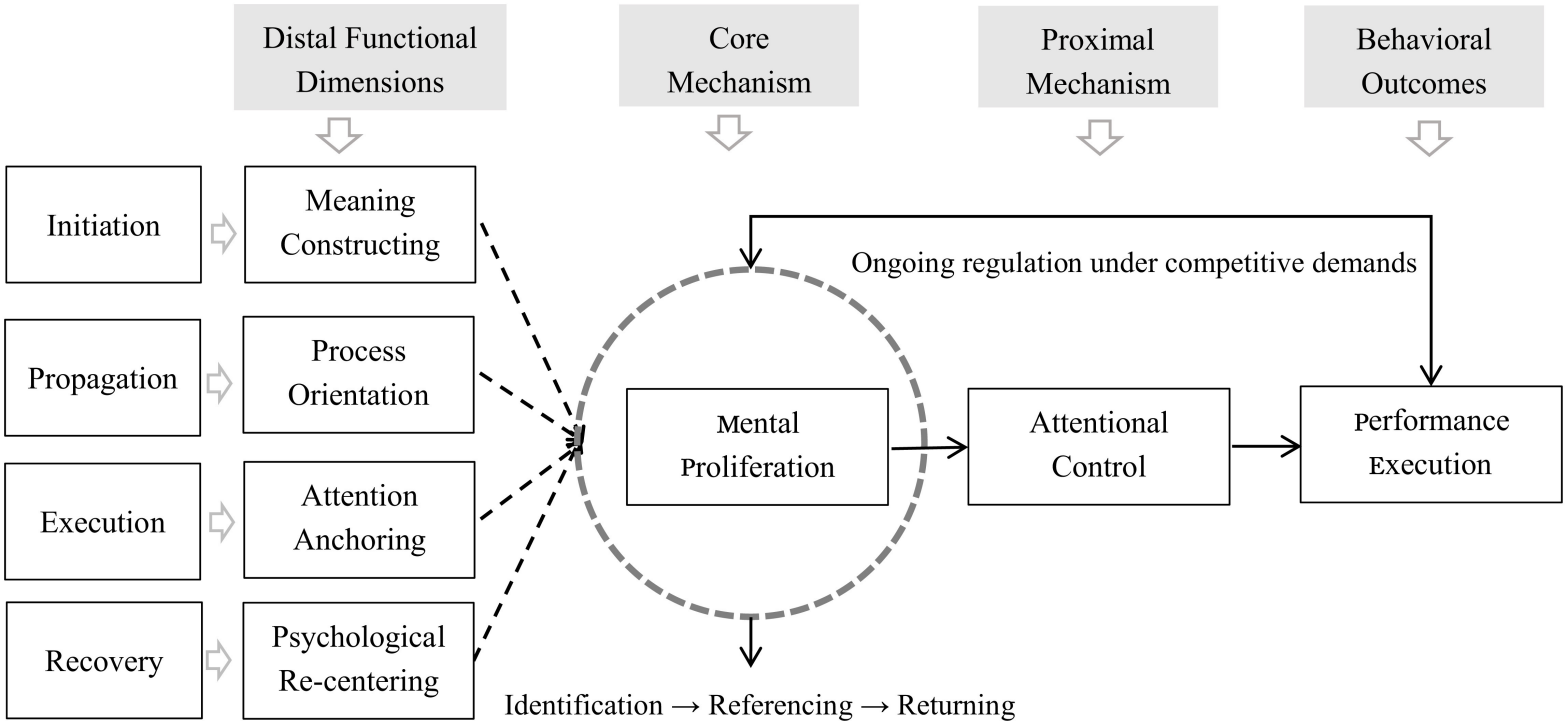
Closed-loop model of psychological regulation.

Attentional regulation, in this context, is not limited to the mere maintenance of focus, but is instead understood as an emergent property arising from the coordination of multiple processes. The model explicitly specifies (a) how regulatory functions are distributed across hierarchical levels, and (b) how these functions are organized within a closed-loop system (Figure 1).

### 5.1 Attentional control as the proximal mechanism of performance execution

At the most immediate level, attentional control constitutes the proximal mechanism through which psychological regulation directly influences athletic performance. Attentional control governs the allocation, stabilization, and updating of perceptual and cognitive resources required for skilled action. Attentional stability directly determines movement timing, coordination, and execution accuracy, particularly under conditions of stress and uncertainty.

### 5.2 Mental proliferation as the core mechanism disrupting attentional control

At the mid-level, attentional control is not an isolated psychological capacity but is continuously shaped by ongoing mental proliferation, which constitutes a core mechanism that undermines attentional stability in competitive settings. When mental proliferation intensifies, attentional resources become increasingly captured by outcome concerns, self-monitoring, emotional reactivity, or future-oriented control attempts. This process disrupts the coordination between top-down control and bottom-up sensory processing, rendering attention unstable and decoupled from performance-critical information. Thus, mental proliferation does not impair performance directly; rather, it leads to performance decrements by destabilizing the attentional processes required for effective execution.

### 5.3 Distal regulatory dimensions as constraints on mental proliferation

To explain how mental proliferation is regulated in competitive contexts, the present model proposes four distal regulatory dimensions: Meaning Construction, Process Orientation, Attention Anchoring, and Psychological Re-centering. These dimensions do not operate at the level of moment-to-moment execution; rather, they function as higher-order constraints that shape how consciousness engages with internal experience.

Meaning Construction regulates value-based proliferation by reducing the tendency to attach excessive symbolic significance to identity, external evaluation. Process Orientation constrains future-oriented striving and retrospective self-evaluation by stabilizing awareness in the unfolding action itself. Psychological Re-centering refers to the successful restoration of functional awareness following attentional drift, while Attention Anchoring provides stable perceptual or proprioceptive reference points that ground attention in task-relevant information. Together, these dimensions limit the initiation, escalation, and persistence of mental proliferation before it disrupts attentional control.

From a process perspective, the four regulatory dimensions can be understood as operating across successive phases of mental proliferation. At the initiation stage, Meaning Construction constrains the assignment of excessive significance to performance events, thereby reducing the likelihood that proliferation is triggered. During propagation, Process Orientation limits the continuation and escalation of task-irrelevant cognitive activity, preventing the expansion of internally generated thought. At the level of execution, Attention Anchoring stabilizes attentional allocation by maintaining a tight coupling between attention and task-relevant cues. Finally, in the recovery phase, Psychological Re-centering enables the detection of disruption and the rapid restoration of functional engagement. Together, these dimensions form a temporally organized regulatory system that intervenes at different stages of the proliferation process while maintaining functional continuity across performance.

### 5.4 The role of the reference chain in dynamic regulation

The model does not assume that mental proliferation can be eliminated or that attentional stability can be maintained continuously. Instead, regulation is conceptualized as an iterative process mediated by a reference chain consisting of identification, referencing, and returning. This chain enables athletes to detect the onset of mental proliferation, re-establish a functional reference point, and restore task-centered awareness. The reference chain operates across all four regulatory dimensions, providing a dynamic mechanism through which deviations are recognized and corrected in real time.

### 5.5 Closed-loop operation of the regulatory system

Taken together, these components form a closed-loop regulatory system. Distal regulatory dimensions shape the baseline orientation of consciousness and constrain mental proliferation; mental proliferation, when unregulated, destabilizes attentional control; attentional instability impairs performance execution; performance demands and internal feedback, in turn, trigger further regulatory responses via the reference chain. Through repeated cycles of disruption and restoration, the system continuously recalibrates itself in response to fluctuating internal and external conditions. In this way, psychological regulation in competitive contexts is not a linear transfer from mental state to performance outcome, but an ongoing, closed-loop process that sustains functional awareness under pressure.

Rather than aiming to establish or maintain idealized mental states, the model emphasizes the ongoing capacity to return to functional awareness during performance. This closed-loop structure reflects both the realities of competitive environments and the Chan Buddhist view that regulation is realized through repeated non-proliferation in the midst of action.

## 6 Discussion

The present model advances mindfulness research in sport by offering a hierarchical and functionally specified account of psychological regulation in competitive contexts. Existing MBIs mechanism models in sport have largely relied on broad experiential constructs which capture important subjective changes, however, they provide limited explanatory power regarding how regulation unfolds during performance execution. By distinguishing between a proximal mechanism (attentional control), a core mechanism (mental proliferation), and distal regulatory dimensions, the present model clarifies the internal organization of mindfulness-related regulation. A key theoretical contribution lies in conceptualizing psychological regulation as a closed-loop system rather than a linear transfer from improved mental states to performance outcomes. This perspective aligns more closely with the dynamic nature of competitive environments, where attention is repeatedly disrupted and must be re-stabilized under conditions of pressure, uncertainty, and time constraint. The introduction of the reference chain further specifies how regulatory processes operate continuously through cycles of identification, referencing, and returning, offering a process-level account that is largely absent from current sport psychology models.

### 6.1 Sport-context variability in regulatory targets

The proposed framework should be understood as describing general mechanisms of performance regulation, while the specific cues and situational triggers involved in each distal functional dimension may vary depending on the different sports. For instance, in closed-skill sports such as shooting or archery, mental proliferation often arises from internally generated cognitive interference. In such contexts, attention anchoring is more likely to be directed toward cues intrinsic to skill execution, such as movement rhythm or key technical elements (Bortoli et al., 2012). By contrast, in open-skill sports such as basketball or football, proliferation may be more frequently triggered by rapidly changing environmental stimuli, including opponent actions and unpredictable game dynamics. Consequently, attention anchoring in these sports tends to be directed toward task-relevant external cues (Chuang et al., 2013). Temporal characteristics of performance may also influence the relative importance of certain regulatory processes. In endurance sports, where performance unfolds over extended periods and is often accompanied by fatigue-induced cognitive drift, psychological re-centering may play a particularly important role by enabling athletes to repeatedly restore attentional engagement during prolonged effort.

At the same time, other dimensions of the framework-particularly Meaning Construction and Process Orientation-are expected to operate as foundational regulatory mechanisms across sport types, as they stabilize motivational orientation and reduce non-attached value-driven cognitive proliferation regardless of the specific performance context.

### 6.2 Individual differences in baseline regulatory capacity

Although the present framework does not explicitly incorporate individual difference variables as structural components, it is important to acknowledge that athletes may differ in the neural systems associated with attentional regulation. Importantly, these neural systems are not fixed, but can be systematically shaped through mindfulness training, in which structured practices (e.g., focused attention on the breath and monitoring of present-moment experience) constitute core operational components. Neuroimaging studies consistently indicate that mindfulness training is associated with functional alterations across multiple large-scale brain networks. for example, the posterior cingulate cortex (PCC) and medial prefrontal cortex (mPFC), which are typically implicated in self-referential processing (Shi and He, 2020), are thought to play a role in reducing self-focused mental activity during meditation (Lutz et al., 2016). In parallel, regions such as the dorsal anterior cingulate cortex (dACC) and anterior insula—commonly associated with salience monitoring and executive attention (Seeley et al., 2007)—are likely to facilitate the regulation and allocation of attentional resources in meditative states (Lutz et al., 2013). Additionally, the striatum, a structure frequently linked to reward-related processing (Lichtenberg et al., 2017), may be involved in supporting regulatory functions during mindfulness practice (Kirk and Montague, 2015).

Taken together, these neural alterations also provide a basis for understanding how individual differences may manifest at the level of baseline regulatory functioning. Athletes with more efficient functional organization within front oparietal control networks may exhibit greater baseline stability in attention anchoring, whereas those with more sensitive salience network dynamics may demonstrate a lower threshold for detecting attentional deviations and initiating re-centering processes. Similarly, reduced baseline dominance of default mode network activity may correspond to a diminished propensity for task-irrelevant self-referential processing, thereby facilitating more rapid recovery from cognitive interference.

From this perspective, mindfulness training—operationalized through repeated engagement in attentional and monitoring practices—may not only induce state-level regulatory effects but also gradually recalibrate the baseline functioning of these neural systems. Such baseline shifts, in turn, influence the efficiency and accessibility of the regulatory processes specified in the present framework. Accordingly, individual differences in performance under pressure may be more appropriately understood as variations in baseline neurocognitive readiness for regulation.

Accordingly, future research may explore how individual differences in baseline neural system functioning influence the operation of the proposed regulatory mechanisms, as well as how targeted training may modify these capacities over time. Such work would provide a basis for extending the present mechanism-oriented framework toward more individualized models of performance regulation.

### 6.3 Toward a multi-method framework for empirical operationalization

To address the issue of empirical operability, the present framework can be extended and tested through a combination of qualitative and quantitative multi-method validation approaches.

On the one hand, at the level of quantitative operationalization, future research may develop standardized measurement tools to examine the relationships among key psychological variables. For example, drawing on (Maddock et al. 2020) approach of assessing mental proliferation through worry and rumination, a mental proliferation Scale can be developed by integrating measurement approaches used to assess maladaptive cognitive processes in sport contexts. In parallel, an Attention Control Scale can be constructed by combining measures of mindfulness in sport settings (Thienot et al., 2014; Zhang et al., 2017) with general-domain assessments of attentional maintenance (Judah et al., 2014). On this basis, longitudinal structural equation modeling based on multi-wave data collection can be employed to examine the statistically inferred causal relationships among mental proliferation, attentional control, and athletic performance. In addition, a Meaning Construction Scale may be developed with reference to existing measures such as the equanimity scale (Sahdra et al., 2010), while a Process Orientation Scale may be constructed based on the 2 × 2 achievement goal framework (Conroy et al., 2003) and prior work by (Jackson and Roberts 1992). These measures can then be incorporated into structural equation models to test the influence of upstream regulatory structures (i.e., meaning construction and process orientation) on downstream processes (i.e., attention anchoring and psychological re-centering).

On the other hand, given the dynamic nature of the reference chain, temporally sensitive methods are required. The Experience Sampling Method (ESM) can be employed to capture real-time fluctuations, whereby brief event-contingent assessments administered during breaks in competition can be used to measure perceived attentional distraction, intrusive thoughts, or declines in task focus. In addition, eye-tracking technology and physiological indicators such as heart rate variability (HRV) can be integrated to assess dynamic changes in attentional control. Furthermore, an interview protocol focusing on athletes' psychological regulation processes may be adopted to capture the actual implementation characteristics of the reference chain.

## 7 Conclusion

This study introduces a process-based, closed-loop model of psychological regulation that reconceptualises performance under pressure as a dynamic process of mental proliferation. By specifying four functionally distinct regulatory dimensions—Meaning Construction, Process Orientation, Attention Anchoring, and Psychological Re-centering—the model delineates how disruption emerges and is regulated across successive stages of performance. Central to this framework is the reference chain (Identification-Referencing-Returning), conceptualized as a recursive control mechanism that enables continuous recalibration. In doing so, the model shifts the field toward a process-level understanding of real-time regulation. This perspective establishes a theoretically integrative and empirically tractable foundation for advancing mechanism-focused research in sport performance.

## Data Availability

The raw data supporting the conclusions of this article will be made available by the authors, without undue reservation.
